# Green synthesis of zinc oxide nanoparticles using waste *Allium cepa* (onion) peel extract and their ROS-mediated cytotoxic activity against A549 lung cancer cells

**DOI:** 10.3389/fchem.2026.1825119

**Published:** 2026-06-17

**Authors:** Rajesh Nayak, ​ Kajol, Kavita Rawat, Nutan Rani, Sapna Yadav, Swati Rani, Kalpana Gupta, Anju Shrivastava, Kalawati Saini, Dipak Maity

**Affiliations:** 1 Department of Chemistry, Miranda House, University of Delhi, New Delhi, India; 2 Department of Zoology, University of Delhi, North Campus, New Delhi, India; 3 Department of Chemistry, Raj Rishi College (RRBM University), Alwar, Rajasthan, India; 4 Integrated Nanosystems Development Institute, Indiana University Indianapolis, Indianapolis, IN, United States; 5 Department of Chemistry and Chemical Biology, Indiana University Indianapolis, Indianapolis, IN, United States

**Keywords:** A549 lung cancer cells, Allium cepa extract, anticancer activity, cytotoxicity, green synthesis, ROS, ZnO nanoparticles

## Abstract

This study reports the green synthesis, characterization, and cytotoxic evaluation of zinc oxide (ZnO) nanoparticles. ZnO nanoparticles have been synthesized using 3 mL of waste peel extracted from Allium cepa (onion) and designated as O3. Comprehensive characterization has been performed using XRD, UV–Visible, FTIR, FESEM, HRTEM, DLS, and fluorescence spectroscopy. The UV–Visible spectrum exhibited an absorption peak at 345 nm, confirming ZnO nanoparticle formation. XRD analysis shows a wurtzite hexagonal phase, while FESEM images show rod-shaped morphology with an average length of ∼400 nm and a width of ∼140 nm. FTIR spectra indicated the presence of functional groups in coating of the nanoparticles surface. The efficacy of these nanoparticles for anticancer activity has been evaluated using MTT, crystal violet assay, AO/PI dual staining, flow cytometry, intracellular ROS estimation, and p53 immunocytochemistry. The MTT assay shows dose- and time-dependent cytotoxicity with an IC_50_ value of 192.63 μg/mL. Crystal violet staining reveals reduced cell density and loss of adherence in treated group. Intracellular ROS estimation using DHE staining shows significant superoxide generation in nanoparticles-treated cells. Flow cytometry demonstrated prominent G1 phase arrest in treated A549 cells, which correlates with increased nuclear p53 expression observed by immunocytochemistry, supporting activation of the p53-dependent checkpoint pathway known to regulate G1 arrest and apoptosis under oxidative stress conditions. Together, these results provide mechanistic support for the apoptosis-inducing effect of the biosynthesized ZnO nanoparticles in A549 lung cancer cells.

## 1 Introduction

Around the world, cancer cases are increasing at an alarming rate. Therefore, there is a need to investigate innovative and reliable therapies for cancer treatment. Traditional treatments for cancer involve radiation therapy, and chemotherapy which commonly encounter several disadvantages like medication resistance and tissue toxicity. Therefore, developing novel cancer treatments that may solve these concerns is desperately required.

The field of nanomedicine continues to develop and is thought to be an efficient and successful sustainable cancer treatment approach. Using metal oxide nanoparticles is one of the most novel approaches to combating cancer. Having their nanoscale characteristics, metal oxide nanoparticles are sometimes described as recent, structured medications that offer constantly released therapeutic material and enhanced medicinal efficacy. For example, several metal oxide nanoparticles have been reported for their anticancer application (Badawy et al., 2023; Satarzadeh et al., 2024; Sundaresan et al., 2023; Waghchaure and Adole, 2023).

Minimizing harmful substance use, eliminating waste, and reducing energy consumption during any chemical reaction are the main criteria of plant-assisted synthesis. In recent years, plenty of literature has been available regarding the anticancer activity of plant-assisted synthesized metal oxide nanoparticles. Manimaran has used the aqueous extract of the mushroom for the synthesis of FeO nanoparticles (Manimaran, 2023). The authors studied the anticancer efficacy of these mushroom extract-based FeO nanoparticles on MG-63, human bone cancer cell lines. The IC_50_ value was reported to be 55.63 μg/mL. Elbrolesy et al. used fruit juice of *Solanum Lycopersicum* (SL) to synthesize ZnO nanoparticles and studied their anticancer activity on HePG2 (hepatocellular carcinoma) and (WI-38) human lung fibroblast cell line (Elbrolesy et al., 2023).

The biocompatible, safe, and non-toxic nature of green synthesized ZnO nanoparticles is responsible for their application in the biomedical field. Therefore, ZnO nanoparticles are a promising alternative to cancer treatment. There are numerous reports highlighting the use of plant-based materials for the synthesis of ZnO nanoparticles. For example, *Scoparia Dulcis* leaf extract, *pinus brutia* leaves extract, *Hardwickia binata* leaves extract, *Pisonia Alba* leaf extract and many more have been utilized for the green synthesis of ZnO nanoparticles (Ismail et al., 2023; Manimegalai et al., 2023; MuthuKathija et al., 2023; Sivasankarapillai et al., 2023).

Scientists have taken a keen interest in the utilization of these green synthesized ZnO nanoparticles in cancer treatment, and numerous studies have recently been published highlighting the anticancer efficacy of these ZnO nanoparticles. Shilajit aqueous extract has been used for the synthesis of ZnO nanoparticles, and Perumal et al. investigated the anticancer activity of these nanoparticles in HeLa cancer cell lines (Perumal et al., 2024). The strong antiproliferative effect of green synthesized ZnO nanoparticles on human colorectal cancer cell lines, namely SW480, Caco-2, and HT-29 was demonstrated by Efati et al. in a dose-dependent way (Efati et al., 2023). The green synthesis of ZnO nanoparticles was reported with the extract of *Lepidium sativum* L. in an aqueous medium. Furthermore, the RT-PCR study revealed the induction of apoptosis was reported through altering the expressions of Bcl-2, Bax, and p53 apoptotic genes. The aqueous seed extract of *Caesalpinia crista* was utilized by Donga et al. for the synthesis of ZnO nanoparticles (Donga and Chanda, 2022). The authors studied the anticancer efficacy of these ZnO nanoparticles on different cancer cell lines, namely normal fibroblast cells, MCF-7, and HeLa cell lines. Recently, Velmani et al. studied the anticancer effect of *Cleome gynandra-*mediated ZnO nanoparticles on A549 cancer cell lines. The authors reported a dose-dependent decrease of the lung cancer lines (*in vitro* study) with an IC_50_ value of 4,000 μg/mL by the ZnO nanoparticles (Velmani et al., 2024). According to the study results, ZnO nanoparticles have noteworthy anti-cancer characteristics when administered at 50–100 μg/mL dose. In another study conducted by Hussain et al., the anticancer effect of *Pandanus odorifer* leaf extract-mediated ZnO nanoparticles was studied on different cancer cell lines, namely A549, MCF-7, and Hep G2 cancer cell lines (Hussain et al., 2019).

The most environmentally benign way to synthesize ZnO NPs is through biological synthesis. Additionally, the synthesis process becomes environmentally friendly and greener if it is mediated by waste materials like vegetable waste. A regular vegetable waste produced in every household, particularly in a nation like India, is onion waste. Onion processing generates a significant amount of byproducts and waste, yet these materials are often discarded, despite being rich in phytochemicals and medicinal compounds. Considering the growing demand for environmentally friendly practices, food processing byproducts such as waste onion peels can be collected and repurposed as ingredients for nutritional supplements and pharmaceutical medications. Research has also shown that onion peel, with quercetin as its main phenolic component, contains a higher concentration of phytochemicals than the edible part of onion (Marefati et al., 2021). However, onion peels showed lower levels of protein (0.88%), ash (0.39%), and crude fiber (0.15%), according to a separate investigation that also highlighted their high carbohydrate content (88.56%) (Ifesan, 2017). Onion peels provide numerous therapeutic benefits, including preventing cancer, obesity, diabetes, neurological diseases, cardiovascular disorders, and microbiological damage. These benefits are attributed to the presence of various bioactive phytochemicals (Kumar et al., 2022).

Considering the challenges posed by drug resistance in cancer treatment and the rapid advancements in nanomedicine research, we utilized zinc oxide (ZnO) nanoparticles to investigate their anticancer activity on A549 lung cancer cell lines. Therefore, we have attempted to synthesize the ZnO nanoparticles using an aqueous waste peel extract of *Allium cepa* (onion) to study the antitumor efficacy on human lung carcinoma A549 cell lines.

## 2 Experimental section

### 2.1 Materials

Onion peels were collected from the canteen of Miranda House College, University of Delhi. Zinc sulfate heptahydrate (ZnSO_4_.7H_2_O) and sodium hydroxide (NaOH, 96%) flakes were purchased from Central Drug House (P) Ltd–CDH, India. Dimethyl sulpho-oxide (DMSO) and methanol were obtained from SRL, India. The chemicals and reagents were all analytical grades and utilized without additional purification. The Human lung adenocarcinoma (A549) cell lines were obtained from the National Cell Repository of Animal Cells (NCCS), Pune, India. MTT (3-(4,5-dimethylthiazol-2-yl)-2,5-diphenyltetrazolium bromide), DMEM (Dulbecco’s modified Eagle’s medium) and dihydroethidium were purchased from Sigma Aldrich, USA. Propidium iodide (PI), Acridine orange (AO), RNase, and crystal violet was purchased from Bangalore Genei. Fetal bovine serum (FBS) was obtained from Invitrogen. p53 primary antibody was purchased from Santa Cruz Biotechnology and secondary antibody was purchased from Invitrogen.

### 2.2 Preparation of 50% onion peel extract

The leftover/waste onion peels were repeatedly cleaned with double-distilled water to get rid of any sticky substances and dust. After cleaning, the peels were allowed to air dry for a few hours at room temperature (RT) before being finally dried for 2 hours at 60 °C in the oven. Peels were ground into a powder using a mixer grinder once they had dried. After that, 50 g of dried peel powder was added to 100 mL of deionized water (0.5 g/mL) taken in a beaker. The beaker contents were heated at 40 °C for 30 min on a hot magnetic plate. Temperature for extraction should not be very high or very low. As high temperatures can decompose phytochemicals, low temperatures can decrease solvation of phytochemicals into the solvent. Therefore, we choose a mild extraction temperature of 40 °C. We have also avoided lengthy heating to prevent decomposition of phytochemicals (Chan et al., 2017). After heating, the contents of the beaker were cooled at room temperature and filtered using Whatmann filter paper (number 1). The obtained onion peel extract was used for the synthesis of ZnO nanoparticles.

### 2.3 Synthesis of ZnO (O3) nanoparticles

We have synthesized ZnO nanoparticles using 3 mL of onion peel extract and named them as O3 nanoparticles. A solution of zinc sulfate (0.2 M) was prepared in a glass beaker using double distilled water. To this solution, 3 mL of as-prepared peel extract was added followed by dropwise addition of 1M NaOH solution. The beaker containing the resultant mixture solution was heated on a magnetic hot plate at 40 °C for 2 h with constant stirring. After cooling to room temperature, the resultant white precipitate of ZnO NPs (O3) was washed with 50% ethanol 4 times. Finally, the washed ZnO NPs were dried in an oven for 8 h at 80 °C to obtain dry powder. The obtained ZnO powders were stored in a glass container for further characterization.

### 2.4 Characterization of ZnO (O3) nanoparticles

The field emission electron microscope (FESEM, JSM-6610LV, JEOL Inc, Japan) was used to assess the morphological characteristics of ZnO (O3) nanoparticles. The High-resolution transmission electron microscope (JEOL, JEM-F200, TEM resolution at 200 kV with cold Field Emission Gun) was also used for high sensitivity surface and electronic structure studies. X-ray diffraction (XRD) pattern was recorded using an X-ray source (Cu Kα, λ = 1.54056 Å) in the 2θ range of 20^o^ to 80^o^ and a 0.02°/s scan rate using D8 Discover, Bruker diffractometer. FTIR spectrum was obtained in the wavelength range of 4,000–400 cm^-1^ using FTIR, 55-Spectrometer, Bruker, USA in ATR mode to identify the various functional groups. The UV-visible absorption spectrum was obtained using UV-vis, Agilent Cary 5,000, R 928 Photomultiplier, USA in the 200–800 nm wavelength range. A fluorescence emission spectrum was obtained using Cary Eclipse equipment and the Cary WinFLR software. Dynamic light scattering particle size distribution and zeta potential analysis were performed using Malvern Zetasizer Nano instrument, UK.

### 2.5 Evaluation of anticancer activity of ZnO (O3) nanoparticles

#### 2.5.1 Cell culture

Human lung adenocarcinoma (A549) cells were cultured in Dulbecco’s Modified Eagles’s Medium (DMEM) supplemented with 10% (v/v) fetal bovine serum (FBS) and maintained at 37 °C in a humidified environment with 5% CO_2_. Cells were sub cultured every 2–3 days using standard trypsinization procedure.

#### 2.5.2 MTT and crystal violet (CV) assay

Cell viability was determined using the MTT assay. Living cells have functional mitochondrial dehydrogenase enzyme, which cleaves the tetrazolium ring to transform yellow soluble MTT into purple insoluble formazan precipitate. A549 cells were seeded at a density of 5 × 10^4^ cells per well in 96-well plates and allowed to be attached overnight. Both ZnO synthesized with chemical wet method and O3NPs were prepared as stock suspensions at a concentration of 1 mg/mL by dispersing the measured nanoparticles in sterile medium (Rani et al., 2022a). The desired working concentrations were obtained through dilution.Cells were treated with different concentrations of ZnO (O3) nanoparticles (100, 150, 200, and 250 μg/mL) for 24 h and 48 h. Untreated cells served as control. Following treatment, 20 µL of MTT solution (5 mg/mL in PBS) was added to each well containing 100 µL of serum-free medium, resulting in a final MTT concentration of approximately 0.83 mg/mL. Plates were incubated for 4 h at 37 °C in the dark. Following incubation, the medium was carefully discarded and 100 µL of DMSO was added to dissolve the formazan crystals. Absorbance was measured at 570 nm using a microplate reader (Epoch™, BIO-TEK, USA). % cell viability was calculated using the equation:

$$\left(\%\right)\text{ cell viability}=\left(\text{OD value of treated sample}/\text{ × }\text{OD value of control sample}\right)\;*100$$

Cell morphology and adherence were assessed using crystal violet staining. A549 cells were seeded in 6-well plates and allowed to be attached overnight. Cells were treated with the IC_50_ concentration of ZnO (O3) nanoparticles for 48 h. Following treatment, cells were gently washed with PBS and fixed with 100% methanol for 1 min at room temperature. The fixation step was performed prior to staining to preserve adherent cells. Cells were then stained with crystal violet solution, followed by washing to remove excess dye, and images were captured for analysis.

#### 2.5.3 Acridine orange/propidium iodide (AO/PI) dual staining

A dual AO/PI staining technique was employed to determine the impact of ZnO NPs on enabling A549 cells to undergo apoptosis. In brief, A549 cells were plated into six-well plates and allowed to adhere overnight in the incubator. Next, for 48 h, the cells were treated to the IC_50_ concentration of as-synthesized ZnO (O3) NPs. The percentage of PI-positive cells was quantified by analyzing fluorescence images using ImageJ software and calculating the ratio of PI-positive cells to total cells.

#### 2.5.4 Flow cytometry analysis

The measurement/quantification of DNA content was done using flow cytometry. It is a popular technique for studying various stages of the cell cycle. The cell cycle study was conducted using a BD Accuri C6 cytometer flow cytometer. ZnO NPs (O3) at an IC_50_ dosage were applied to A549 cells for 48 h. Specifically, 1 × 10^6^ cells were taken out and resuspended in phosphate-buffered saline (PBS). After centrifuging the mixture to obtain a pellet, 70% cold ethanol in PBS was used to fix the cells. Following a 60-min incubation period at −20 °C, samples were twice washed with PBS and then re-incubated in a staining solution comprising 50 µL of RNase solution and 200 µL of PI. Lastly, samples were once more incubated for 30 min at room temperature (RT) in the dark.

#### 2.5.5 Intracellular ROS measurement by DHE staining

Intracellular reactive oxygen species (ROS) generation was assessed using dihydroethidium (DHE) staining. A549 cells were seeded in 96-well culture plate and allowed to adhere overnight. Cells were treated with cisplatin, ZnNPs, onion extract (OE), and onion-derived nanoparticles (O3NPs) for 8 h, while untreated cells served as control. Following treatment, cells were washed with PBS and incubated with DHE (final concentration: 10 µM) in serum-free medium for 15 min at 37 °C in the dark. After incubation, cells were washed gently with PBS three times to remove excess dye. Nuclei were counterstained with DAPI. Cells were immediately observed under fluorescence microscope (Nikon Eclipse Ti2R). Images were captured at 20× magnification. Mean fluorescence intensity was quantified using ImageJ software. Statistical analysis was performed using GraphPad Prism software.

#### 2.5.6 Immunocytochemistry for p53 localization

p53 expression and nuclear localization were analyzed by immunofluorescence staining. A549 cells were grown on sterile coverslips and subjected to different treatments (cisplatin, ZnNPs, OE and O3NPs) for 16 h. After treatment, cells were washed with PBS and fixed with 4% paraformaldehyde for 15 min at room temperature. Cells were then washed and permeabilized using 0.1% Triton X-100 in PBS for 10 min. Non-specific binding was blocked by 5% BSA in 0.1% PBST for 1 h at room temperature. Cells were incubated with primary anti-p53 antibody (catalogue: sc-99; Santa Cruz Biotechnology) overnight at 4 °C. After washing with 0.05% PBST 3X for 10 min each, cells were again incubated with blocking buffer for 1 h at room temperature. Then, cells were incubated with secondary antibody (goat-anti mouse IgG Alexa fluor 488 from Invitrogen # catalogue: A11029) for 1.5 h in the dark. Next, cells were washed with PBST for 30 min. Nuclei were counterstained with DAPI, and coverslips were mounted using antifade mounting medium. Fluorescence images were captured using Nikon Eclipse Ti2R at 20× magnification with constant exposure settings. Nuclear localization was determined based on co-localization of p53 fluorescence with DAPI nuclear staining.

#### 2.5.7 Statistical analysis

All biological experiments were performed in triplicate, repeated thrice and the data are presented as mean ± standard deviation (SD). Statistical analysis was carried out using GraphPad Prism (version 8.0.1). Prior to analysis, data distribution was assessed for normality. Differences between multiple groups were evaluated using one-way analysis of variance (ANOVA), followed by Dunnett’s *post hoc* test to compare treatment groups with the control. The p-value of less than 0.05 is considered statistically significant. IC_50_ values were determined using non-linear regression analysis (dose–response curve fitting) in GraphPad Prism.

## 3 Result and discussion

### 3.1 X-ray diffraction (XRD)

**FIGURE 1:**
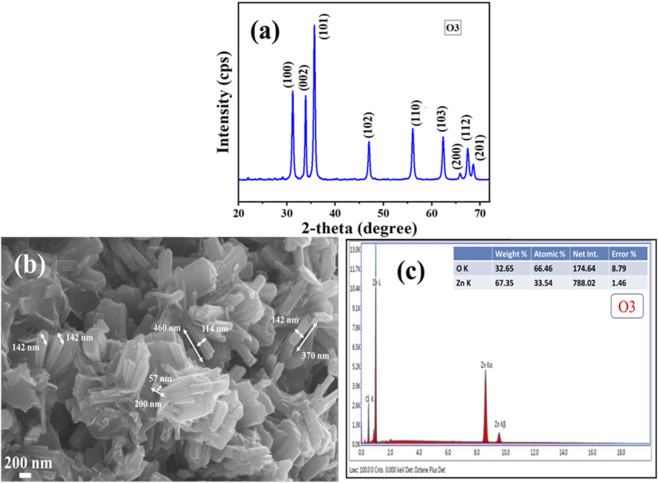
**(a)** X-ray diffraction (XRD) pattern of ZnO (O3) nanoparticles **(b)** FESEM images of synthesized ZnO (O3) nanoparticles **(c)** Energy Dispersive Spectroscopy (EDS) of synthesized ZnO (O3) nanoparticles.

The XRD pattern of biosynthesized ZnO (O3) NPs is given in Figure 1a. The resulting XRD pattern matches with the wurtzite structure (hexagonal phase; JCPDS # 36-1451) (Gupta et al., 2024; Saputra et al., 2024). The ZnO (O3) NPs depicted peaks corresponding to (100), (002), (101), (102), (110), (103), (200), (112), and (201) hkl planes. Also, the sharp and intense peaks confirm that the as-synthesized ZnO (O3) NPs have an ideal crystalline structure. Moreover, the absence of an impurity peak indicates that synthesized ZnO NPs have phase purity (i.e., no secondary phase is present).

### 3.2 Field emission scanning electron microscopy (FESEM) and energy dispersive spectroscopy (EDS)

The morphological analysis of synthesized ZnO (O3) nanoparticles has been performed using field emission scanning electron microscopy (FESEM). Figure 1b shows field emission scanning electron micrographs of synthesized O3 nanoparticles wherein, rod shape morphology of synthesized nanoparticles obtained. The average width was ∼142 nm and length ranged from 370 to 420 nm. The observed atomic % of Zn and O elements for O3 nanoparticles are 33.54% and 66.46% respectively from the EDS analysis plot given in Figure 1c. Similar rod shape morphology has been described for green synthesized ZnO nanoparticles (Dhanalakshmi et al., 2016; Mohamed et al., 2019).

### 3.3 High-resolution transmission electron microscopy (HRTEM)

**FIGURE 2:**
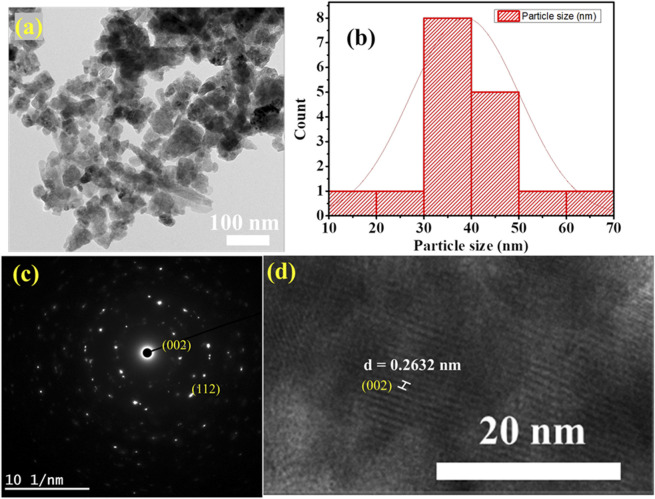
**(a)** HRTEM micrograph, **(b)** histogram from HRTEM image **(c)** selected area electron diffraction (SAED) pattern, and **(d)** interlayer d-spacing images of synthesized ZnO nanoparticles.

We have performed high-resolution transmission electron microscopy (HRTEM) analysis to investigate the surface morphology and crystalline structure of the synthesized ZnO nanoparticles. Figure 2a confirms the successful synthesis of ZnO nanoparticles. Average size of synthesized ZnO nanoparticles is obtained 38 nm from the HRTEM data which is shown in Figure 2b. The selected area electron diffraction (SAED) pattern of the ZnO nanoparticles is illustrated in Figure 2c, where the presence of concentric circles indicates their crystalline nature of synthesized nanoparticles. Additionally, we measured the inter-planner distance (d-spacing) of the (002) hkl plane to be 0.2632 nm, as depicted in Figure 2d. Our HRTEM findings are consistent with previously reported data (Agarwal et al., 2019; Bagabas et al., 2013; Jamdagni et al., 2018). Overall, the HRTEM analysis confirms both the synthesis of nanosized ZnO particles and their crystalline properties.

### 3.4 UV-visible spectroscopy

**FIGURE 3:**
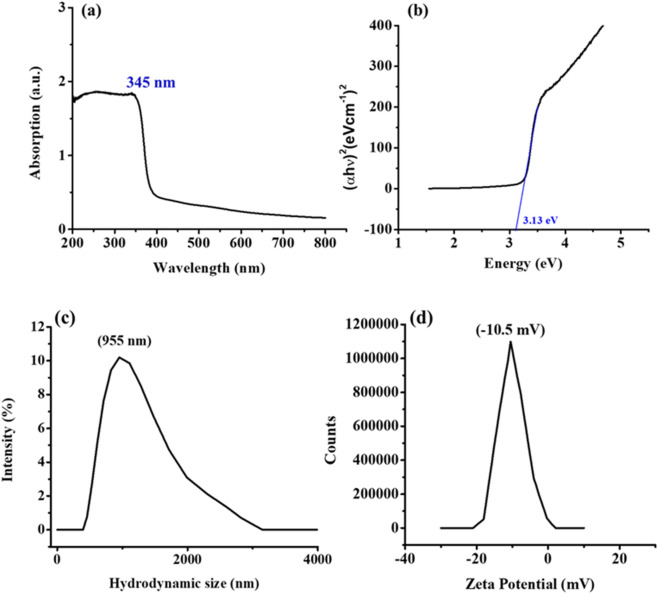
**(a)** UV-Visible absorption spectrum of ZnO (O3) nanoparticles, **(b)** Tauc plot (αhν)^2^ vs. hν of synthesized O3 nanoparticles, **(c)** Hydrodynamic size distribution curve of the of ZnO (O3) nanoparticles, and **(d)** Zeta potential curve of ZnO (O3) nanoparticles.

UV-visible spectroscopy was utilized to analyze and measure the band gap of the synthesized O3 nanoparticles. The absorption spectrum of the as-synthesized ZnO (O3) nanoparticles and the corresponding Tauc plot are shown in Figures 3a,b. The UV-Vis spectrum reveals that the absorption maximum (λmax) occurs at 345 nm, confirming the successful synthesis of ZnO nanoparticles. This absorbance peak is attributed to the intrinsic band gap of ZnO, which is a result of electron transitions from the valence band to the conduction band. Several researchers have reported similar positions for the absorbance peaks of ZnO nanoparticles (Al Awadh et al., 2022; Murali et al., 2023; Yassin et al., 2022).

The direct band gaps of the synthesized O3 nanoparticles were determined using Tauc’s relation (Equation 1). Equation 1 is also mentioned in the literature (Islam et al., 2024; Saini et al., 2022).

αhv1γ=Ahv−Eg (1)

Herein Equation 1, hν is the photon’s energy, A is a constant, E.g., is the band gap energy and γ is 2 (for allowed indirect transition) or ½ (for allowed direct transition). The energy band gap value (E.g.,) for O3 nanoparticles was measured to be 3.13 eV, which is comparable to the reported band gap energy value of 3.20 eV for ZnO nanoparticles (Ruvalcaba-Manzo et al., 2025). Similar, energy band gap values of 3.14 eV, 3.17–3.19 eV and 3.35 for ZnO nanoparticles have been reported in the literature (HabtemariamT et al., 2026; Mediou et al., 2022; Rizvi et al., 2025).

### 3.5 Dynamic light scattering (DLS)

The DLS studies were conducted to determine the hydrodynamic size and zeta potential of synthesized ZnO (O3) nanoparticles. The hydrodynamic size distribution curve is presented in Figure 3c. The measured hydrodynamic size of the synthesized ZnO (O3) nanoparticles is 955 nm, which can be attributed to the agglomeration of these nanoparticles. The HRTEM micrograph in Figure 2a confirms this agglomeration. The likely reason for the agglomeration is the presence of surface-attracting phytochemicals on the surface of the ZnO (O3) nanoparticles (Rani et al., 2022b). Rani et al. reported a high hydrodynamic size of ZnO nanoparticles at 580 nm (Rani et al., 2022b). The zeta potential curve for the synthesized ZnO (O3) nanoparticles is shown in Figure 3d. The observed zeta potential for these nanoparticles is −10.5 mV. A negative zeta potential value less than −30 mV indicates that the ZnO nanoparticles exhibit high colloidal stability, which enhances their potential for biomedical applications (Rani et al., 2022c; Saini et al., 2022). Yassin et al. reported a zeta potential of −7.45 mV for biosynthesized ZnO nanoparticles using pomegranate peel extract, which also demonstrated effective antifungal activity (Yassin et al., 2022).

#### 3.5.1 FT-IR analysis

**FIGURE 4:**
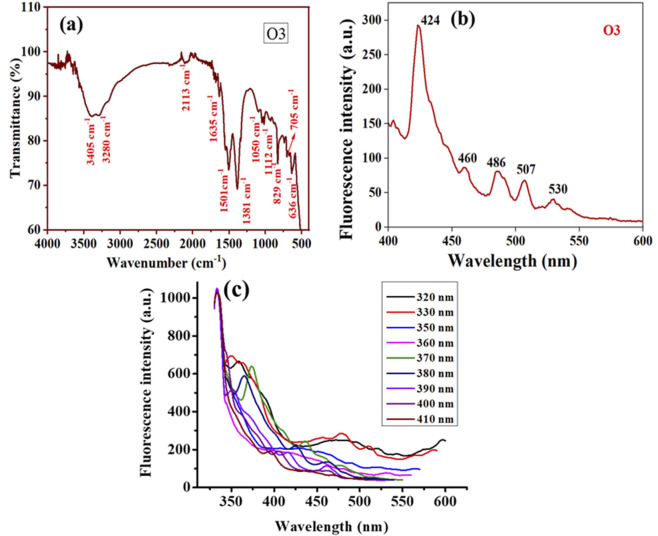
**(a)** FT-IR spectrum of synthesized ZnO (O3) nanoparticles **(b)** the Fluorescence emission spectrum of the synthesized ZnO (O3) nanoparticles with an excitation wavelength of 340 nm, and **(c)** the Fluorescence emission spectra of the synthesized ZnO (O3) nanoparticles with excitation wavelengths of 320 nm, 330 nm, 350 nm, 360 nm, 370 nm, 380 nm, 390 nm, 400 nm, and 410 nm respectively.

FTIR spectroscopy is used to detect the presence of phytochemical coating adhered to the surface of biosynthesized ZnO nanoparticles. Figure 4a presents the FT-IR spectrum of ZnO (O3) nanoparticles. The observed bands at 3,405 cm^-1^ and 3,280 cm^-1^ are due to O-H stretching bending. The peaks at 1635 cm^-1^ are due to N-H bending vibrations of amides and amine groups. In addition, the obtained peaks between 1381 cm^-1^ and 1050 cm^-1^ show the presence of C-O stretching and C=C stretching bands (MuthuKathija et al., 2023). The peaks at 829 cm^-1^, 705 cm^-1^, and 636 cm^-1^ are allocated to metal-oxygen bond (Almarhoon et al., 2022). Thereby, the presence of these peaks confirms the formation of ZnO nanoparticles. Such findings suggest that naturally occurring substances such as flavonoids and phenolic compounds may be utilized as reducing and stabilizing agents for the formation of ZnO nanoparticles. Islam et al. have also investigated the presence of phytochemicals such flavonoids, alkaloids, carboxylic acid, and polyphenols in the ZnO-NPs synthesized coated with *Allium cepa* L. extract (Islam et al., 2024).

### 3.6 Fluorescence analysis

Figures 4b,c display the fluorescence emission spectra of the biosynthesized ZnO (O3) nanoparticles (NPs). All emission profiles were recorded at room temperature in HPLC water. Figure 4b illustrates the fluorescence emission spectrum of the biosynthesized ZnO (O3) NPs when excited at a wavelength of 340 nm. The spectrum shows peaks at 424 nm, 460 nm, 486 nm, 507 nm, and 530 nm, confirming the formation of pure ZnO nanoparticles. Estrada-Urbina et al. reported a similar fluorescence spectrum for ZnO nanoparticles (Estrada-Urbina et al., 2018). The observed fluorescence spectrum indicates that the blue emission peak at 424 nm is due to the formation of zinc interstitial defects, while the strong blue emission at 460 nm and the faint blue-green emission at 486 nm are attributed to oxygen vacancy defects (Goh et al., 2026). The emission peaks at 507 nm and 530 nm, related to antisite defects, are associated with deep-level or trap-state emissions. These peaks arise from transitions from the deep donor level (oxygen vacancies in ZnO) to the valence band. Figure 4c shows the emission spectra in the wavelength range of 400–600 nm at different excitation wavelengths: 320 nm, 330 nm, 350 nm, 360 nm, 370 nm, 380 nm, 390 nm, 400 nm, and 410 nm. Notably, the emission peaks remain consistent across these excitation wavelengths, indicating that the molecule emits light from the same excited state for all these excitation wavelengths.

### 3.7 Evaluation of green synthesized ZnO (O3) nanoparticles on the viability of A549 cells

**FIGURE 5:**
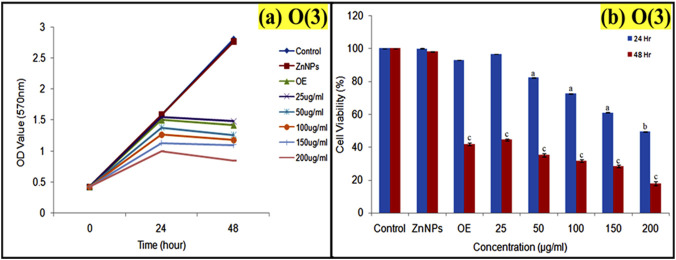
**(a)** Depicts the treatment of A549 cells with O3-NPs at the mentioned concentrations and time duration. Data was represented in triplicate by performing three separate experiments; **(b)** depicts the cell death induction by O3 NPs in A549 cells determined by MTT assay. Data are expressed as mean ± SD of three independent experiments. Bars sharing the same letter are not significantly different, whereas different letters (a, b, c) indicate statistically significant differences (p ≤ 0.05).

ZnO nanoparticles (O3) were evaluated for their impact on the viability of A549 cells using the MTT assay. Different concentrations of ZnO (O3) nanoparticles (25 μg/mL, 50 μg/mL, 100 μg/mL, 150 μg/mL, 200 μg/mL) were used to investigate the impact on cell survival. The optical density (OD) value was measured using the microplate reader at 570 nm. The higher the OD value, the more living cells are indicated. The absorbance value at two distinct time intervals (24 and 48 h) represented the number of viable cells. Figure 5a depicts the cell viability graph for ZnO (O3) nanoparticles. Figure 5b represents the cell death induction by O3 NPs in A549 cells investigated by MTT assay. Moreover, a noticeable reduction in cell viability was evident after a 48-h incubationperiod. The calculated IC_50_ value for O3NPs (192.63 μg/mL) was observed to be close to the highest tested concentration (200 μg/mL). While this concentration range allowed for an approximate estimation of cytotoxic potency, it may limit the precision of IC_50_ determination. The IC_50_ value is presented with a 95% confidence interval, calculated from triplicate measurements to reflect variability in the data. Future studies should include a broader concentration range extending beyond 200 μg/mL (e.g., up to 400–500 μg/mL) to obtain a more accurate dose–response curve and IC_50_ value. Nonetheless, the current data clearly demonstrate a dose-dependent cytotoxic effect of O3NPs. Son et al. reported an IC_50_ value of 156.26 μg/mL for A549 cell lines with guava leaf extract-mediated ZnO nanoparticles (Son et al., 2023). Also, Velmani et al. reported IC_50_ of 4,000 μg mL^-1^ for the *Cleome gynandra* extract-mediated ZnO nanoparticles against A549 cancer cell lines (Velmani et al., 2024).

### 3.8 Effect on the morphology of A549 cells by green synthesized ZnO (O3) nanoparticles

**FIGURE 6:**
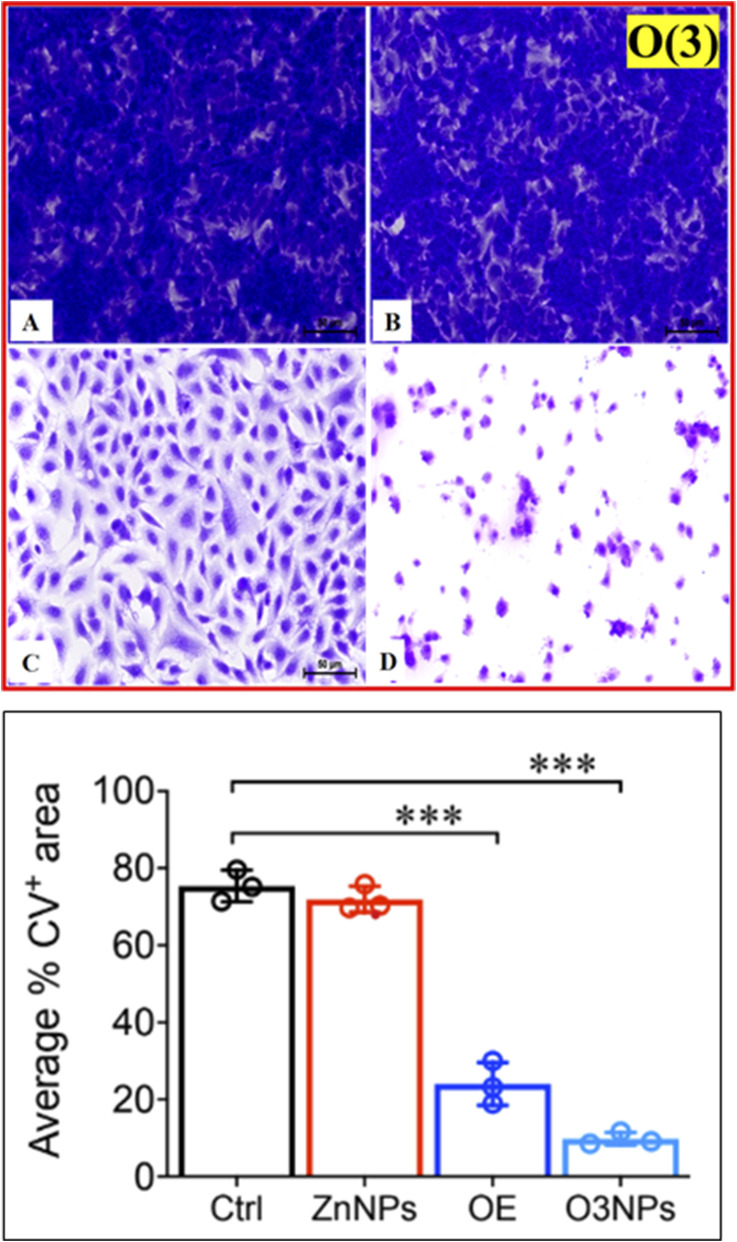
Represents the morphology of ZnO (O3) NPs treated human lung cancer A549 cells. **(A)** No treatment (Control group) **(B)** Cells treated with ZnO- NPs synthesized using wet chemical method (ZnO nanoparticles) (reported earlier (Rani et al., 2022a) **(C)** cells treated with onion peel extract, **(D)** Cells treated with ZnO NPs synthesized from peel extract (O3 NPs) with corresponding IC_50_ dose, at magnification-×200. Bar graphs represent the average percentage of crystal violet-positive stained area (%CV area), reflecting cell viability, quantified using ImageJ. Data is represented as mean ± SD, (p ≤ 0.001 ***). OE: Onion extract; ZnO nanoparticles: chemically synthesized zinc oxide nanoparticles; O3NPs: onion extract-mediated zinc oxide nanoparticles synthesized in the present study.

Crystal violet staining was used to determine how the shape/morphology of A549 cells was affected by green synthesized ZnO (O3) nanoparticles. The crystal violet stained micrographs of A549 cells treated with ZnO (O3) nanoparticles are represented in Figure 6. The A549 cells were stained with crystal violet and after that examined under an upright microscope (Nikon) at 200× magnification. The cells were treated with an IC_50_ dose (192.63 μg/mL) for 48 h. Our analysis of the crystal violet micrographs shows a decrease in cell number, along with rounded shapes, poor adherence, and significant cell shrinkage.

### 3.9 Induction of apoptosis in A549 cells by green synthesized ZnO (O3) nanoparticles

**FIGURE 7:**
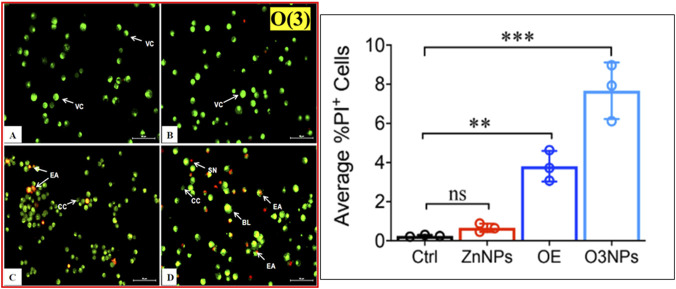
Apoptosis and Morphological analysis of A549 cells double stained with AO and PI as observed under a fluorescent microscope (Nikon). **(A)** Cells without treatment, **(B)** Cells treated with ZnO- NPs synthesized with chemical-wet method (ZnO nanoparticles) (reported earlier (Rani et al., 2022a)), **(C)** Onion peel extract treated cells, **(D)** Cells treated with ZnO NPs synthesized from peel extract (O3 NPs) with corresponding IC 50 dose (where EA: early apoptosis; VC: viable cells and SN: secondary necrosis. The percentage of PI-positive cells was quantified by analyzing fluorescence images using ImageJ software and calculating the ratio of PI-positive cells to total cells. Data is represented as mean ± SD, (p ≤ 0.01 **, p ≤ 0.001 ***).

Apoptosis was confirmed by examining specific changes in the morphological characteristics of the cells. These changes include nuclear or cytoplasmic fragmentation, chromatin condensation, and cell shrinkage. To ensure the induction of apoptosis in A549 cells, we conducted dual-labeling fluorescent experiments using acridine orange (AO) and propidium iodide (PI). AO staining allowed us to visualize healthy cells or early apoptotic cells with fragmented DNA, while PI staining highlighted dead cells. The AO/PI fluorescent micrographs are shown in Figure 7 for the green-synthesized ZnO (O3) nanoparticles. We treated A549 cells with an IC_50_ dose of 192.63 μg/mL of ZnO (O3) nanoparticles to perform the AO/PI dual labeling experiments. Subsequently, we examined the A549 cells under a Nikon fluorescent microscope to assess viable cells and identify early and late stages of apoptosis. We observed signs of early apoptosis. Notably, we noticed early apoptosis, secondary necrosis, and chromatin condensation in O3-treated A549 cells. These were the indications of cell death. Viable cells were verified after 48 h by the green color and undamaged nuclei of untreated A549 cells. When A549 cells were exposed to green-synthesized ZnO (O3) nanoparticles, the appearance of these morphological alterations/modifications verified the induction of cell death/apoptosis.

### 3.10 Cell cycle analysis by flow cytometry

**FIGURE 8:**
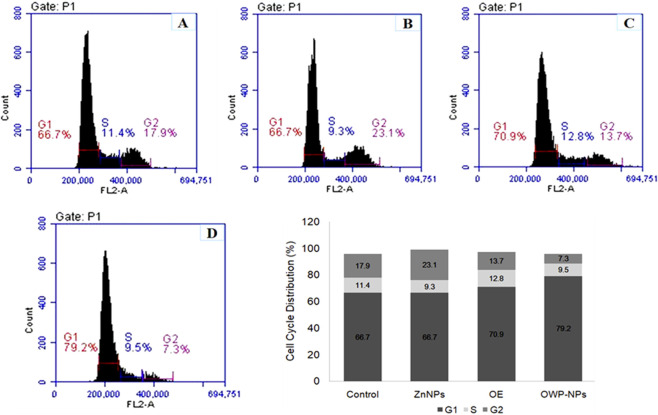
Flow cytometry analysis of cell cycle arrest in A549 cells treated with **(A)** 193 μg/mL of O3 NPs for 48 h. The percentage distributions of the cell cycle shown by the bar diagram. **(A)** Cells without treatment, **(B)** Cells treated with ZnO NPs synthesized with chemical-wet method (ZnNPs), **(C)** Onion extract treated cells, **(D)** Cells treated with ZnO NPs synthesized from peel extract (O3-NPs) with corresponding IC_50_ dose.

Cell cycle analysis was performed to assess the impact of green-synthesized ZnO (O3) nanoparticles on the cell cycle pattern. In addition, flow cytometry analysis was conducted to examine the distribution of cell cycle phases in A549 cells treated with ZnO (O3) nanoparticles. The impact on the progress of the cell cycle was investigated following a 48-h exposure to an IC_50_ dose of ZnO (O3) nanoparticles. Figure 8 demonstrates the results of flow cytometry analysis. Also, % of cell cycle phase arrest by the ZnO (O3) nanoparticles was given in Table 1.

**TABLE 1:** Shows the cell cycle phase arrest by the green synthesized zinc oxide (O3) nanoparticles.

S. No.	Cell cycle phase	Untreated control	ZnO (O3)
01.	G1 phase	66.7%	79.2%
02.	S phase	11.4%	9.5%
03.	G2 phase	17.9%	7.3%

### 3.11 Intracellular ROS generation in A549 cells

**FIGURE 9:**
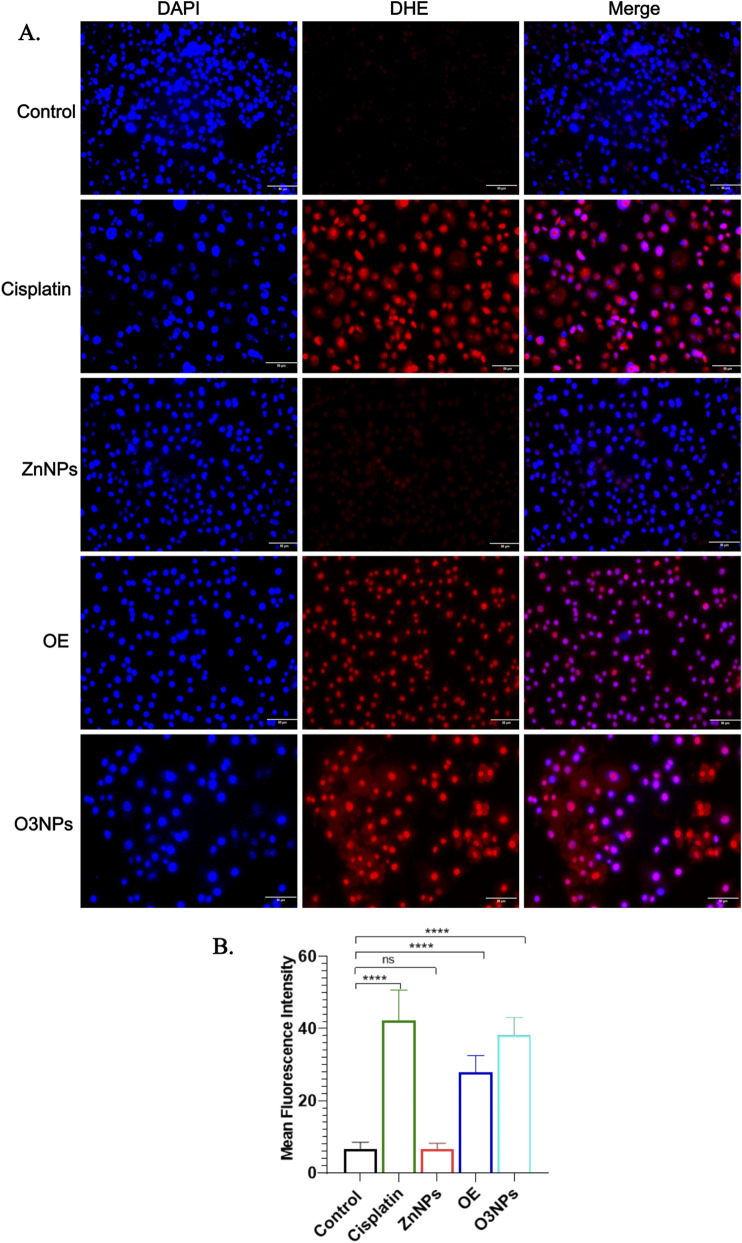
Intracellular ROS generation in A549 cells measured by DHE staining. **(A)** Representative fluorescence microscopy images of A549 cells showing intracellular reactive oxygen species (ROS) levels detected using dihydroethidium (DHE, red) after different treatments: Control, Cisplatin, ZnO nanoparticles, OE (Onion extract), and O3NPs. Nuclei were counterstained with DAPI (blue). Merged images represent the overlay of DAPI and DHE channels. Increased red fluorescence indicates elevated ROS production in treated groups compared to control. Images were captured at ×200 magnification. Scale bar = 50 µm. **(B)** Quantification of mean fluorescence intensity (MFI) of p53 signal signal across treatment groups. Data are expressed as mean ± SD. Statistical analysis was performed using one-way ANOVA (****p < 0.0001; ns, not significant).

Oxidative stress is widely recognized as a key mechanism behind the cytotoxic effects of ZnO nanoparticles (Akhtar et al., 2012). In this study, we evaluated intracellular reactive oxygen species (ROS) levels in A549 cells after various treatments using DHE fluorescence staining. Cisplatin was included as a positive control due to its well-documented ability to induce ROS-mediated oxidative stress in cancer cells (Marullo et al. 2013). Fluorescence microscopy revealed low basal ROS levels in the untreated control group, as indicated by weak red fluorescence (see Figure 9A). In contrast, cells treated with cisplatin, onion extract, and nanoparticles synthesized from onion extract exhibited significantly higher DHE fluorescence, suggesting increased ROS production. However, the group treated with chemically synthesized ZnO nanoparticles did not show a noticeable increase in ROS levels compared to the control group.

Quantitative analysis of mean fluorescence intensity, presented in Figure 9B, revealed a significant increase in ROS levels in the cisplatin, onion extract, and O3NPs groups compared to the control. Conversely, the ZnO nanoparticles group remained statistically non-significant (one-way ANOVA, p < 0.05).

This observation clearly shows that the production of reactive oxygen species (ROS) is mainly influenced by the bioactive components in the onion extract coating, rather than by the ZnO core itself. This finding highlights a significant synergistic interaction between the ZnO nanoparticles and the plant-derived coating, which likely contributes to the enhanced biological activity of the onion extract-coated ZnO nanoparticles (O3NPs) compared to chemically synthesized ZnO nanoparticles.

### 3.12 p53 localization in treated A549 cells

**FIGURE 10:**
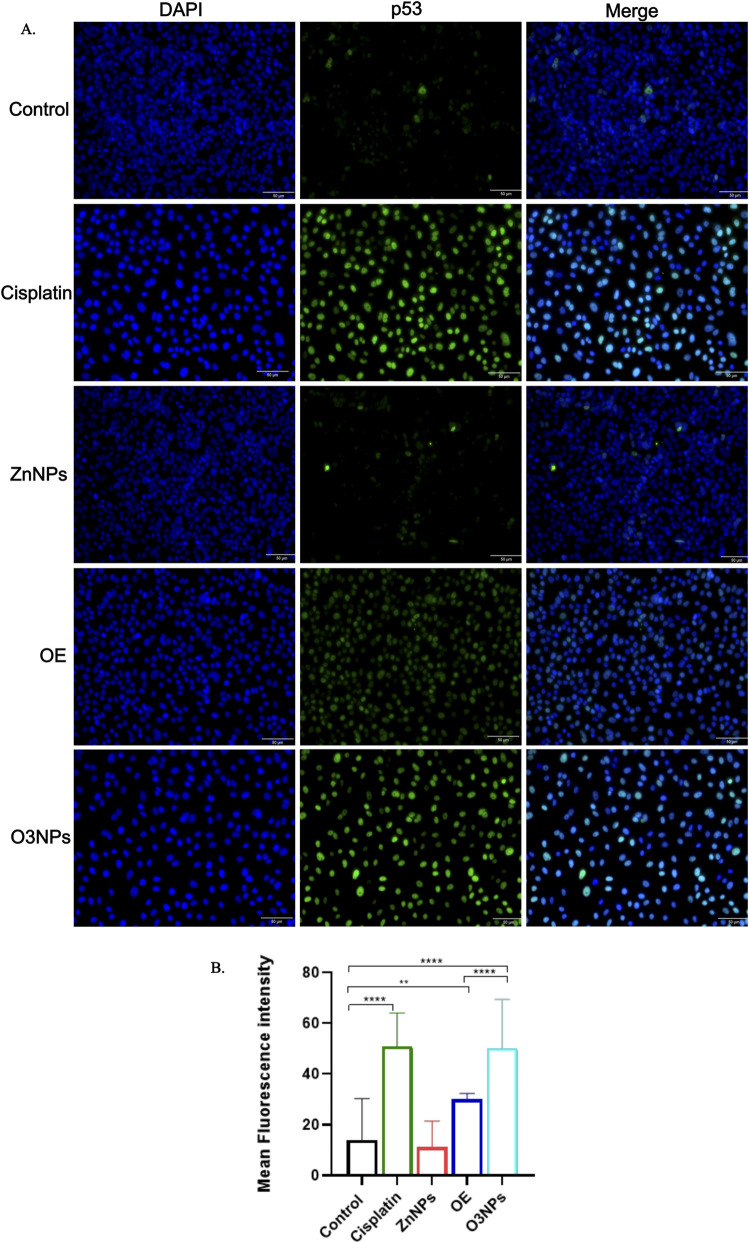
Nuclear localization of p53 in A549 cells by immunofluorescence. **(A)** Representative immunofluorescence images of A549 cells showing p53 localization after different treatments: Control, cisplatin, ZnO nanoparticles, OE (onion extract) and O3NPs. Cells were immunostained with a primary anti-p53 antibody followed by a fluorescent secondary antibody (green) and nuclei were counterstained with DAPI (blue). Merged images indicate nuclear localization of p53 based on overlap of green signal with DAPI- stained nuclei. Cisplatin, OE and O3NPs treatments show enhanced nuclear p53 staining compared with control, whereas ZnO nanoparticles group shows minimal p53 comparable to control. Images were captured at ×200 magnification. Scale bar = 50 µm. **(B)** Quantification of mean fluorescence intensity (MFI) of p53 signal across treatment groups. Data are expressed as mean ± SD. Statistical analysis was performed using one-way ANOVA (****p < 0.0001; **p < 0.01).

p53 immunocytochemistry was performed to detect nuclear p53 accumulation, which is known to regulate cell cycle arrest and apoptosis through downstream targets such as p21 and pro-apoptotic factors like Bax and Puma in response to oxidative DNA damage (Luo et al., 2022). However, p21 expression was not directly evaluated in the present study. Immunofluorescence analysis demonstrated treatment-dependent changes in p53 localization, as shown in Figure 10. Control cells showed weak p53 staining. Cisplatin and O3NPs treatments produced strong nuclear p53 localization, as evidenced by co-localization with DAPI staining. OE treatment showed increased nuclear p53 signal compared with control, but weaker than that observed with O3NPs. The ZnO nanoparticles group displayed minimal p53 staining similar to control cells. These findings indicate that O3NPs induce a stronger p53 nuclear response than OE alone.

### 3.13 Limitations of the study

This study has several limitations that should be acknowledged. First, the findings are based solely on *in vitro* experiments, which may not fully replicate the complexity of *in vivo* biological systems. Furthermore, mechanistic insights into the cellular pathways involved were not extensively explored. Future studies involving *in vivo* models and broader dose ranges are recommended to validate and extend these findings.

## 4 Conclusion

In conclusion, ZnO nanoparticles were successfully synthesized using the waste peel extract of *Allium cepa* (onion) in an environmentally friendly manner. The onion peel extract functions as both a capping agent and a reducing agent. Various advanced techniques, such as Field Emission Scanning Electron Microscopy (FESEM), Powder X-ray Diffraction (XRD), Fourier Transform Infrared Spectroscopy (FTIR), Dynamic Light Scattering (DLS), High-resolution transmission electron microscopy (HRTEM), Ultraviolet-Visible Spectroscopy (UV-Vis), and fluorescence spectroscopy, were utilized to characterize the ZnO (O3) nanoparticles. The synthesized ZnO (O3) nanoparticles exhibited a rod-shaped morphology and displayed a hexagonal wurtzite phase. Notably, the anticancer activity of these nanoparticles was investigated using A549 cancer cell lines. The O3 nanoparticles demonstrated dose- and time-dependent cytotoxicity (IC_50_ = 192.63 μg/mL at 48 h), morphological changes, induction of apoptosis, and arrest at the G1 phase (79.2%). This process was facilitated by the generation of reactive oxygen species (ROS) and the translocation of the p53 protein into the nucleus. The results from various anticancer studies suggest that green-synthesized ZnO (O3) nanoparticles have the potential to be a strong anticancer agent in the biomedical field.

## Data Availability

The raw data supporting the conclusions of this article will be made available by the authors, without undue reservation.
